# Connecting the dots: Neutrophils at the interface of tissue regeneration and cancer

**DOI:** 10.1016/j.smim.2022.101598

**Published:** 2021-10

**Authors:** Emma Nolan, Ilaria Malanchi

**Affiliations:** Tumour Host Interaction Laboratory, The Francis Crick Institute, 1 Midland Road, NW1 1AT London, United Kingdom

**Keywords:** Neutrophils, Tissue repair, Cancer

## Abstract

Knowledge about neutrophil biology has exponentially grown over the past decades. A high volume of investigations focusing on the characterization of their initially unappreciated multifaceted functions have grown in parallel with the immunity and the cancer fields. This has led to a significant gain in knowledge about their functions not only in tissue defence against pathogens and the collateral damage their overactivation can cause, but also their role in tissue repair and regeneration especially in the context of sterile injuries. On the other hand, the cancer field has also intensively focused its attention on neutrophil engagement in the many steps of the tumorigenic process. This review aims to draw the readers’ attention to the similar functions described for neutrophils in tissue repair and in cancer. By bridging the two fields, we provide support for the hypothesis that the underlying program driving cancer-dependent exploitation of neutrophils is rooted in their physiologic tissue protection functions. In this view, cross-fertilization between the two fields will expedite the discovery of therapeutic interventions based on neutrophil targeting or their manipulation.

## 1 Introduction

Upon tissue injury, wound healing represents the physiological reaction that restores tissue homeostasis. Neutrophils are the first cells to be recruited at the site of injury, promoting the inflammatory response that mediates immune cell recruitment, clearance of debris and/or invading microbes. The subsequent resolution of this inflammatory phase triggers the onset of the reparative phase, whereby cellular proliferation, angiogenesis and remodelling of the extracellular matrix restores organ functionality. Neutrophil activity as the first line of defence against infectious agents is well established, and their key functions in acute infections is highlighted by the severe immune deficiency caused in humans by neutrophil insufficiency. More recently, our understanding of their functions and diverse roles in tissue repair has increased and a general key role in the maintenance of tissue homeostasis has emerged [1,2]. Our knowledge of neutrophil biology has exponentially increased over the recent years, emphasizing their previously underestimated plasticity. In a recent Review [3], we have defined neutrophil functional heterogenicity based on three parameters: *space*, *time* and *disease context*. These respectively refer to the ability of neutrophils to respond to local signals and to adapt their properties (spatial parameter); to the fact that neutrophils can be released early before full maturation into the circulation, and that, even when released at a mature stage, they change properties over time via an ageing process (temporal parameter); and finally, how different neutrophil behaviours are triggered in response to disease states, for instance their abnormal wound healing function observed in diabetes (disease context).

In cancer, a disease that profoundly alters the physiology of the organism, neutrophil heterogenicity appears to be dramatically perturbed, likely due to the intersection of all three parameters mentioned above. Accordingly, their behaviour is so multifaced that it is hard to reconcile to a clear consensus around a pro-tumoral or anti-tumoral function. Furthermore, a large body of literature conveys a more complex role with a spectrum of context-dependent activity over time and for different tumor types. The dramatic change in the tissue environment during tumor progression undoubtedly contributes to the evolving relationship between neutrophils and cancer over time. Thankfully, their recognized crucial role in cancer has stimulated intensive studies on neutrophil behaviour in different tumor contexts, exposing the broad spectrum of their activities and greatly expediting our knowledge about their context-dependent regulation.

Importantly, this duality in neutrophil functions in cancer is analogous to the dichotomy observed in wound healing, where numerous studies attest to the beneficial role of neutrophils in tissue repair, yet the collateral damage of neutrophil activities is associated with exacerbated injury [4,5]. Furthermore, like in cancer, heterogeneity in neutrophil subsets has also been reported in various types of tissue injury, although the extent to which this polarisation modulates the outcome of repair is still under investigation.

In this review we will summarise the current knowledge of neutrophils’ tissue repair functions, and their pathologic responses in relation to cancer initiation and outgrowth in the primary or secondary tissue. In doing so, we hope to convey the striking parallelism between the functions of neutrophils in tissue repair and their pro-tumorigenic activity. This raises the possibility that their physiological response to a tissue perturbation guides their response to a growing tumor. Overall, it is our view that an increased understanding of the links between neutrophil functions in injury and cancer inflammation could inform the development of novel therapies to restrain and/or harness the activities of these fascinating cells.

## 2 Neutrophils in tissue injury & repair

**Fig. 1:**
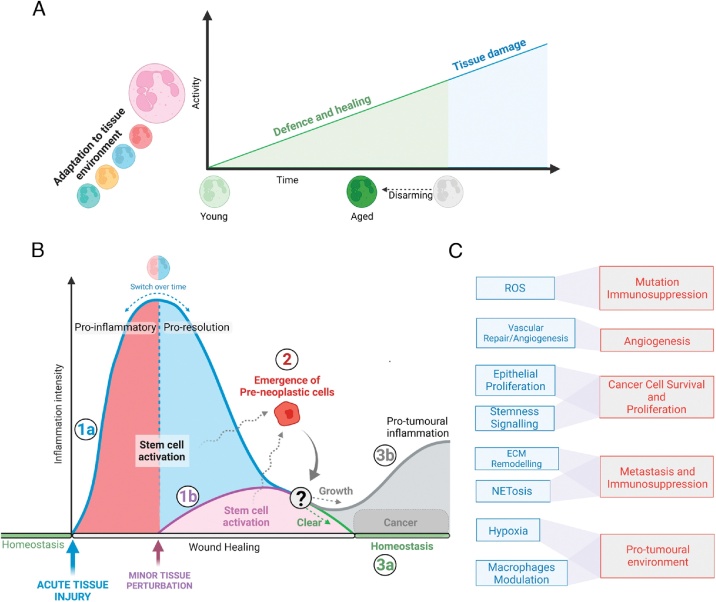
Visual summary of the concepts discussed. The figure was created with BioRender.com. A. Schematic overview of the level of basal neutrophil activity as a function of time. This diagram suggests a model whereby an overly high level of activation might be tissue-damaging, and the process of disarming allows maintenance of optimal neutrophil function. B. Schematic representation of a proposed model for tumor initiation. Starting from an acute tissue injury (1a) or a tissue perturbation (1b), either an acute or a more moderate level of neutrophil responses is initiated. In both cases, a pro-resolution and pro-repair inflammatory status is induced in the tissue. Here, mutated pre-neoplastic cells (2) (which were already present in the tissue or that may arise as consequence of the injury) will be cleared in most cases (3a). Neutrophils will contribute to tissue repair and return to homeostasis. When pre-neoplastic cells are not cleared and manage to grow, a scenario which is still not fully understood, cancer will be initiated (3b). As a tumor cell mass emerges in the tissue, the local environment will change, pro-resolution inflammation will now persist and slowly evolve into pro-tumoral inflammation by engaging with the new emerging tumor microenvironment. Here neutrophils are a major player. This will lead to cancer formation. C. Examples of parallelism between neutrophil tissue repair (blue) and pro-tumoral (red) activities.

It has long been appreciated that tissue insults, both infection-driven or in sterile injuries such as myocardial infarction, are followed by the rapid recruitment of circulating neutrophils to the site of injury via the release of pathogen- and damage-associated molecular patterns (PAMPs and DAMPs, respectively). Mechanical injury to cells following trauma can also release mitochondrial DAMPs, which activates innate immune responses [6]. In the injured tissue, neutrophils phagocytose cellular debris and invading microbes, produce high concentrations of reactive-oxidative species (ROS), and release a myriad of antimicrobial peptides, inflammatory mediators, and proteolytic enzymes. During an infectious insult, neutrophils can also release extracellular traps (NETs), lattices of DNA filaments decorated with toxic enzymes that effectively immobilise and neutralise bacteria [7]. The neutrophil-driven inflammatory response is essential for the elimination of pathogens and initiating tissue repair processes. However, excessive neutrophil activation, NETosis and the persistent release of neutrophil toxic effectors can drive further tissue damage and even harm surrounding healthy tissues [8,9]. As such, uncontrolled inflammation can convert physiological tissue repair and regeneration to pathological tissue damage, and drive the development of chronic diseases and non-healing wounds (Fig. 1a).

More recently however, a beneficial role for neutrophils in tissue repair has emerged, with studies reporting anti-inflammatory, pro-angiogenic and regenerative neutrophil functions [4,5]. This can be mediated directly, via the clearance of cellular debris and the release of growth factors, anti-inflammatory molecules and pro-angiogenic factors, or indirectly via macrophage-mediated phagocytosis of apoptotic neutrophils (efferocytosis). Efferocytosis stimulates macrophage polarisation to a reparative phenotype, triggering the production of pro-repair cytokines including TGF-β and inflammation resolution [10]. Fascinatingly, a neutrophil-intrinsic program of proteome disarming was recently reported to protect tissues against neutrophil-mediated damage [11]. In a process dependent on the chemokine receptor CXCR2, murine neutrophils released from the bone marrow during the night were found to progressively undergo an homeostatic change in their proteome as they aged in the circulation, which coincided with a loss in granule content and decrease of NET-forming capacity. This progressive loss of granules over time was also observed in circulating human neutrophils. Since the authors had previously identified a circadian regulation of neutrophil migration into tissues during the day [12], this study elegantly linked diurnal aging with their potential to cause tissue damage. Neutrophils migrating into tissues over time have a reduced arsenal of toxic cargo, thus were less likely to elicit organ damage in response to endotoxin-induced acute lung inflammation (ALI) compared to freshly released neutrophil in the night [11] (Fig. 1A).

Overall, the outcome of the neutrophil response to injury, either tissue-damaging or tissue-reparative, is almost certainly context-dependent, taking into account the trigger of the initial insult, the local microenvironment of the injured tissue, and the interactions between neutrophils and other resident and/or recruited cells. To add to the complexity, neutrophil heterogeneity contributes to their differential response to tissue injury and repair, which has become particularly evident in studies of cardiovascular injury [13]. In this section, we will briefly review several different injury contexts in which neutrophils play a critical role. We will focus on their emerging functions in tissue repair, their interactions with other cells within the injured site, and their heterogeneity in these specific contexts.

### 2.1 Wound repair in the skin and intestinal mucosa

The bactericidal action of neutrophils is crucial for restricting microbial invasion when the skin or mucosal barrier is breached. Overzealous activation of these potent neutrophil functions and release of their toxic cargo has the potential to cause significant collateral tissue damage, chronic inflammation and impaired wound healing [4]. However, neutrophils also have beneficial functions in the healing of epithelial surfaces [14]. Indeed, neutropenic patients, genetically-modified mice with impaired neutrophil recruitment and mice deficient in the neutrophil protease matrix metalloproteinase 8 (MMP-8) all exhibit diminished skin wound repair [[15], [16], [17], [18]]. In the injured skin, neutrophils secrete an array of cytokines including TNFα and VEGF, that contribute to re-epithelization, angiogenesis and wound closure [19,20]. Recently, neutrophils were shown to cooperate with the commensal skin microbiota and plasmacytoid dendritic cells (pDC) to accelerate skin wound repair [21]. Commensal bacteria colonizing skin wounds trigger neutrophil activation and CXCL10 production, stimulating recruitment of type 1 interferon-producing pDC to the injured skin and accelerating wound closure via activation of fibroblasts and macrophages. Importantly, human neutrophils recruited to skin wounds display a pro-healing transcriptional program compared to circulating neutrophils [22]. This is characterised by the upregulation of genes associated with keratinocyte and fibroblast proliferation, angiogenesis and extracellular matrix (ECM) remodelling. This phenomenon highlights how neutrophils can adapt accordingly to the tissue environment they infiltrate and increase their heterogeneity to functionally tailor their response, in this case, to the process of skin repair. Aging may influence the pro-reparative activity of neutrophils, since increased host age is associated with delayed wound repair in both humans and mice, coinciding with declining neutrophil functions [23,24]. Interestingly, neutrophil depletion by anti-Gr-1 antibody treatment resulted in a more severe delay in wound repair in aged mice compared to younger animals. Perhaps fewer neutrophils are sufficient for wound repair in young mice due to their enhanced functionality compared to aged mice, or there may be compensatory mechanisms that are less active in older animals [25].

Following injury to the intestinal mucosa, rapid neutrophil infiltration is instrumental in preventing bacterial translocation from the intestinal lumen. Massive neutrophil transepithelial migration into the inflamed intestine, however, can lead to epithelial injury and delayed mucosal healing. This is mediated by proteases released by mobilized neutrophils including elastase and MMPs [26,27], as well as neutrophil-derived Junctional Adhesion Molecule-like protein (JAML). JAML binds to the epithelial tight junction protein Coxsackie and Adenovirus Receptor (CAR), compromising the intestinal barrier and decreasing epithelial proliferation [28]. More recently, epithelial injury in the intestine has been attributed to the release of neutrophil-derived microparticles armed with high concentrations of myeloperoxidase (MPO) and MMPs [29], as well as proinflammatory miRNAs [30]. In the latter study, the authors demonstrated that the delivery of miR-23a and miR-155 from neutrophils to intestine epithelial cells promotes the accumulation of double-strand breaks and inhibits DNA repair, exacerbating inflammation and impeding wound recovery. On the other hand, neutrophils can also contribute to the resolution of mucosal wounds. Following transepithelial migration, neutrophil binding to Intracellular adhesion-molecule 1 (ICAM-1) on apical epithelial cells triggered a boost in epithelial proliferation via Akt and B-catenin signalling, and enhanced wound healing in a biopsy-based colonic-mucosal injury model [31]. Furthermore, in a mouse model of colitis, neutrophils undergoing respiratory burst were shown to induce a hypoxic niche within the intestinal epithelium [32]. This led to stabilization of hypoxia-inducible factor-1 (HIF-1) in neighbouring epithelial cells, promoting inflammation resolution. Taken together, a growing body of experimental evidence supports the important contribution of neutrophils in healing of the skin and intestinal mucosa, in parallel to their classic bactericidal properties.

### 2.2 Neutrophils in acute lung injury and repair

Acute lung inflammation (ALI) and its severe form, acute respiratory distress syndrome (ARDS), are characterised by lung edema due to disruption of the alveolar epithelium, increased permeability of the alveolar-capillary barrier, and the impairment of arterial oxygenation. ALI and ARDS are accompanied by the rapid influx of neutrophils into the alveolar space, and can be triggered by pneumonia and sepsis, as well as non-infectious causes including trauma and high-pressure ventilation [33]. There is a multitude of experimental studies and clinical data to suggest that neutrophil migration and activation causes injury to alveolar epithelial cells and barrier disruption (reviewed in [34]). Indeed the concentration of neutrophils in bronchoalveolar lavage (BAL) fluid correlates with the severity and outcome of ARDS [[35], [36], [37]].

Neutrophil-driven lung injury is predominantly attributed to the release of cytotoxic enzymes, NETosis and ROS generation, while in a model of transfusion-induced ALI, PSGL-1-dependent capture of circulating platelets by recruited neutrophils contributed to injury of the pulmonary vasculature [38]. Furthermore, massive neutrophil recruitment and excessive NETosis have recently been shown to be a potent driver of ARDS and immunothrombosis in Covid19 patients [39,40]. As mentioned earlier, in a model of endotoxin-induced ALI, the cell-intrinsic program known as neutrophil-disarming, which moderates the ability of neutrophils to produce NETs and regulates the amount of cytotoxic mediators released during their lifetime in circulation, was shown to induce less damage in the lung during the day [11]. This association between the circadian oscillation of neutrophil function with the severity of lung damage could influence the clinical presentation of lung injury in patients.

Importantly, accumulating evidence also pinpoints a role for neutrophils in alveolar epithelial regeneration and lung re-epithelisation, a pivotal event in the resolution of ARDS. Neutrophil transmigration following intratracheal LPS or keratinocyte chemokine administration resulted in the activation of β-catenin signalling and proliferation in alveolar type 2 cells (AT2), via neutrophil elastase-mediated cleavage of E-cadherin [41]. Mice that received injections of the neutrophil-depleting Ly6G antibody, or neutropenic G-CSF knockout mice, demonstrated reduced AT2 proliferation and impaired re-epithelisation in an acid-induced lung injury model that recapitulates features of ARDS [42]. Unbiased proteomic analysis of BAL fluid from Ly6G-treated mice versus control mice 12 h after lung injury suggested a possible role for matrix metalloproteinases MMP-2 and MMP-9 in epithelial regeneration. In support of this, neutrophil-derived MMP-9 was linked to lung repair in a recent study, with exogeneous MMP-9 treatment significantly improving lung recovery and proliferation in neutropenic mice following ventilator-induced ALI [43]. An improved epithelial wound healing response was also observed *in vitro* when BAL fluid from neutropenic patients with ARDS was supplemented with exogenous MMP-9. In an alternative mechanism, the uptake of neutrophil-derived miR-223 by alveolar epithelial cells attenuated lung inflammation and edema following both infection and ventilation-induced acute lung injury, possibly via repression of the poly(adenosine diphosphate-ribose) polymerase-1 (PARP-1) [44]. Thus, neutrophil infiltration and activation plays an important role in the restitution of lung epithelial integrity upon tissue damage, which in some cases, they might themselves have exacerbated.

### 2.3 Neutrophils in CNS injury and repair

The poorly regenerative capacity of the central nervous system (CNS) can result in chronic neurological deficits following brain, spinal cord, or ocular injury. Therefore, there is an urgent need to develop new therapies that can mitigate and even reverse damage. Although traditionally classified as detrimental in the CNS [45], a beneficial role for neutrophils in CNS injury repair emerged after Ly6G antibody-mediated neutrophil depletion was shown to hamper healing and worsen functional recovery after spinal cord injury in mice [46]. Neutrophils also support regeneration in the optic nerve via secretion of the neurotrophic factor oncomodulin [47], as well as clearance of nerve debris and axonal regrowth after peripheral nerve injury [48,49]. Importantly, a novel neutrophil subset with neuroprotective properties was recently identified using mouse models of optic nerve and spinal cord injury [50]. This Ly6G^lo^CD14^high^ subset, which displayed characteristics of immature neutrophils such as a ring-shaped nucleus and low CD101 expression, promoted axonal regeneration via the secretion of pro-reparative growth factors including NGF and IGF-1. Thus, this work provides another example of neutrophil adaptation enabling a tissue-specialized response.

This dual role for neutrophils in augmenting or repairing damage in the CNS is also evident in studies of ischemic stroke, where neutrophil-derived factors have been linked to secondary injury to the blood-brain-barrier and edema after ischemia (reviewed in [51]). Also, NETs were recently shown to impair neovascularisation and vascular remodelling after stroke [52]. However, in the context of ischemic retinopathy, NETosis-mediated clearance of senescent vasculature stimulated vascular remodelling and regeneration [53]. A pro-reparative subpopulation of neutrophils was also detected in ischemic brain tissue in a rodent model of stroke, with their frequency correlating with increased neuronal survival, improved functional recovery and reduced infarct volume [54,55]. Toll-like receptor 4 (TLR4) appears to be a key modulator of neutrophil polarization in the CNS, with specific myeloid ablation of TLR4 skewing neutrophils towards a neuroprotective phenotype following stroke that correlates with a reduced infarct volume [56].

### 2.4 Neutrophils in cardiovascular repair

The deleterious effects of neutrophils after myocardial infarction (MI) are well-established [[57], [58], [59]], with experimental studies demonstrating that neutrophil depletion or inhibition reduces acute cardiac injury and infarct size [[60], [61], [62], [63]]. However, anti-inflammatory, pro-angiogenic and pro-reparative effects of neutrophils in cardiac injury are also emerging (reviewed in [64,65]). Neutrophil secretion of neutrophil-gelatinase-associated lipocalin (NGAL) triggers macrophage polarisation to a regenerative M2 phenotype with an enhanced efferocytosis ability, a key event in post-MI inflammation resolution [66]. In contrast to earlier studies, the authors showed that neutrophil-depleted mice subjected to MI had a dysregulated healing response, increased fibrosis and a progressive loss of ventricular function.

Importantly, several studies have reported temporal neutrophil polarisation following myocardial infarction. In a landmark study by Ma et al., a first-wave of pro-inflammatory N1 neutrophils were detected in the heart shortly after MI that mediated the clearance of apoptotic cardiomyocytes, whereas a pro-reparative N2 neutrophil subset increased in frequency over time, supporting inflammation resolution and cardiac repair [13]. Comparably, Ly6G^hi^CXCR2^+^ and Ly6G^lo^CCR2^+^ neutrophil subsets have been identified in the MI heart with similar temporal timing as N1 and N2 subsets [67]. A cardiac neutrophil proteome shift over 7 days post-MI has also been reported, with neutrophils at day 7 displaying a reparative signature [68]. It is possible that these studies have identified the same or similar neutrophil subpopulations. More recently, several cardiac neutrophil clusters were identified over time in post-MI mice using single-cell RNA sequencing [69,70]. Of note were the two major subsets identified at days 3 and 5 (SiglecF^hi^ and SiglecF^lo^), with the increase in SiglecF^hi^ neutrophils over time coinciding with the inflammation resolution phase. The authors proposed that the upregulation of SiglecF expression may induce neutrophil apoptosis and thus stimulate macrophage efferocytosis, as has been observed in SiglecF-expressing eosinophils [71]. Interestingly, cardiac healing may be influenced by sex-based differences in neutrophil polarization, with female mice displaying a blunted neutrophil-driven inflammatory response immediately after MI compared with males, and higher levels of anti-inflammatory N2 markers during the repair phase. This correlates with improved survival in female mice [72]. Finally, neutrophil polarisation has also been linked to the pathogenesis of atherosclerosis, the major risk factor for an infarction event. Elevated neutrophils and enhanced NETosis is associated with an increased size and destabilization of atherosclerotic plaques in humans and mice [[73], [74], [75]], which has been linked to the priming and activation of lesion-infiltrating macrophages that secrete IL-1β [75]. However, a recent study reported that while pro-inflammatory neutrophils primed by low-dose LPS challenge exacerbated atherosclerosis in ApoE^−/−^ mice, neutrophils that were re-programmed *ex vivo* to a pro-resolving N2 phenotype were associated with a significantly reduced plaque size [76]. These studies show a promising potential for the exploitation of neutrophil polarisation dynamics for the treatment of atherosclerosis as well as improved cardiac healing after MI.

## 3 Neutrophil fate after injury: reverse transmigration

A critical phase in the resolution of the inflammatory response is the clearance of neutrophils from the injured tissue, indeed neutrophil persistence is associated with further tissue damage and chronic inflammation. While regulated neutrophil apoptosis is a key mechanism to resolve inflammation [77], it is now widely appreciated that neutrophils do not always die at the site of injury and instead can return back to the vasculature [78]. This process, termed reverse transmigration (rTEM), was first reported using infused radiolabelled neutrophils in a model of glomerular capillary injury in rats [79], and directly visualized using in vivo time-lapse imaging in zebrafish larvae [[80], [81], [82]]. In an elegant study that combined intravital imaging and photoactivation, murine neutrophils were visualized re-entering the vasculature following sterile thermal hepatic injury, journeying through the lungs where they upregulated CXCR4 expression, before homing back to the bone marrow where they were eliminated [83]. Importantly, egress from injured tissue to the vasculature was dependent on neutrophil serine protease activity, and neutrophil accumulation in cathepsin C-deficient mice was associated with delayed revascularisation compared to control mice. This suggests reverse migration contributes to tissue repair. Unsurprisingly, given the dichotomy observed with neutrophil function, rTEM has also been reported to contribute to tissue damage. Following ischemia-reperfusion injury, reverse-transmigrating neutrophils were reported to traffic to the lungs and trigger secondary damage, suggesting a possible mechanism by which local inflammation can generate a systemic multi-organ response [84,85]. More recently, injury to the skin following acute UV light exposure was shown to stimulate a neutrophil-dependent inflammatory and injury responses in the murine kidney, with photoactivation confirming a subset of renal neutrophils reverse-transmigrated through the UV-exposed skin [86]. This is of interest clinically, since sunlight exposure is associated with aggravation of systemic disease including nephritis in patient with lupus [87]. These findings are supported by clinical evidence, since neutrophils bearing markers of rTEM were identified in the blood and pulmonary vasculature of patients with acute pancreatitis that developed subsequent acute lung injury [88]. Clearly further studies to delineate the functional implication of neutrophil rTEM in different injury contexts are required. Moreover, it remains to be determined if rTEM broadly occurs and contributes to other pathological condition such as cancer.

## 4 From tissue repair to fostering cancer

### 4.1 Origin of cancer and its link to tissue injury and inflammation

Our current knowledge on the origin of cancer stipulates the requirement for a number of incidents, including (but not limited to) genetic mutations, sustained proliferation, immune evasion and the activation of angiogenesis, which ultimately increase the chance of a growing tumor mass to emerge in a tissue. However, precise knowledge of these key events is still lacking. The fact that somatic mutations typically found as drivers in squamous cell carcinoma can also be found in normal tissue has now re-defined genetic mutations as key events, indicating they alone are not sufficient to generate tumors [89]. Importantly, the fact that mutations are clonally expanding indicates that they occur in tissue stem cells and progenitors. Indeed, a study looking at the acquisition of genetic mutations during life reported a steady accumulation over time and that different tissues have different mutation spectra, probably reflecting their proliferation and turnover period. The study found that driver mutations in cancer show similarities with the mutation spectra of tissue-specific stem cells, suggesting that the intrinsic mutational process correlates with tumorigenesis [90]. Certainly, tissue stem and progenitor cells have been considered the most likely cell of origin of cancer by several studies, even if the level of plasticity within the tissue should be considered [91] as well as that certain sets of genetic drivers can reprogram most epithelial cells toward diverse fates [92]. Importantly, tissue damage, by inducing an activation of stem cell function, was shown to increase the cancer risk of an organ [93]. However, tissue injury does not only trigger tissue stem cells activity, but as discussed earlier, is accompanied by an inflammatory reaction, which has the aim to foster and restore tissue homeostasis.

The relationship between inflammation and cancer is very profound [94]. Indeed, a direct causal link between inflammation and cancer was demonstrated by a large clinical study, the CANTOS trial, where treatment with a monoclonal antibody targeting IL-1β (canakinumab) not only significantly reduced the incidence of recurrent cardiovascular events in the absence of lipid lowering in patients with myocardial infarction compared with placebo, but also had a significant effect on reducing the incidence of lung cancer and overall cancer mortality [95]. A previous large clinical study also demonstrated the efficacy of daily aspirin treatment in lowering the long-term risk of cancer death [96].

According to our current knowledge, cancer arises in the context of a tissue injury or tissue disruption when the presence of a certain set of oncogenic mutations triggers the generation of pre-cancerous cells. What drives those pre-cancerous cells to break the tissue integrity, overcome the local immune-control and to outgrow is not fully understood. However, once this occurs, the presence of an emerging tumor structure leads to a switch in the local tissue environment, from normal to cancerous tissue. With a loss of normal tissue identity, the pursue of tissue repair is lost and the inflammation is diverted toward a program that supports the new abnormal cancerous tissue growth. Here the program of inflammation resolution accompanying tissue repair is not achieved as the original tissue to be restored no longer exists, instead it becomes pro-tumoral inflammation. Cancer inflammation is an integral part of the environment where cancer cells reside, the tumor microenvironment (TME), within an unsteady tumor structure that constantly grows and evolves, where homeostasis does not exist, and inflammation never ceases (Fig. 1B).

### 4.2 Neutrophils and the origin of cancer

As discussed earlier, key events dictating the likelihood of tumor onset are the mutation load in stem and progenitor cells, and their activation status within the context of tissue injury and repair where inflammatory cells are engaged. Neutrophils can contribute to the genetic mutational load in response to tissue exposure to a carcinogenic agent [97] and in the context of acute inflammation [98], which indeed leads to increased tumorigenesis. They were also reported to sustain the pathologic states of chronic inflammation in inflammatory bowel disease (IBD) via induction of double-strand breaks in epithelial cells. Importantly, this impaired resolution of inflammation and led to persistent disease-associated tissue injury in IBD patients, who are at increased risk of colon cancer [30,99].

Tissue stem cell activation is the second important factor determining cancer risk [93]. While a direct link between neutrophils pro-tumorigenic activity and a boost in tissue stem cells activity is not yet demonstrated, an indirect link via the connection between neutrophils and their support of tissue stem cells in tissue regeneration can be identified. Upon lung injury, transmigrating lung neutrophils were found to foster epithelial lung repair by inducing β-catenin signalling in AT2 cells [41]. Intriguingly, Wnt signalling is important not only for lung development and repair, but it was shown to maintain stemness of AT2 cells and their activity during lung post-injury regeneration [100,101]. AT2 cells were also described to function as stem cells that contribute to alveolar homeostasis, repair and cancer [102], while Wnt responder cancer cells, a subset of lung adenocarcinoma, were shown to be crucial in supporting cancer stemness and tumor growth [103]. Therefore, the engagement of neutrophils in tissue repair might unintentionally serve to increase the risk of cancer in the tissue, via an increased mutagenic load and tissue stem cell function.

### 4.3 Neutrophils: from tissue repair to cancer support

Many parallelisms exist between the activities of neutrophils in tissue repair and their role in cancer progression (Fig. 1C). As discussed earlier, neutrophils promote revascularisation after injury via the secretion of pro-angiogenic cytokines including VEGF [104], or through the clearance of senescent vasculature stimulating angiogenic remodeling [53]. Similarly, in cancer, the role of neutrophils in tumor-associated angiogenesis is well established either directly via angiogenic factors or indirectly via ECM remodelling [105]. ROS generation during neutrophil respiratory burst, which is crucial for the clearance of pathogens after injury-mediated barrier disruption, is also implicated in tumor progression and metastasis. This can be attributed to their indirect actions in the TME, since neutrophil ROS release can induce T cell and NK cell immunosuppression [[106], [107], [108], [109]]. The intracellular accumulation of ROS in neutrophils is also linked to NETosis, which is a unique response against pathogens, and now has a recognized role in fostering metastasis [110]. The pro-metastatic function of NETs relies not only on their ability to trap cancer cells in circulation [111], favour their intravasation [112] and drive ECM remodelling via proteases associated with net-DNA [113,114], but even net-DNA can directly induce cancer cell invasion and proliferation via a specific receptor, the transmembrane protein CCDC25, found on cancer cells [115]. Moreover, NETs were reported to contribute to the immune-suppressive function of neutrophils by blocking their interaction with T cells [116].

In addition, many of the cytokines, chemokines and growth factors released by neutrophils during an injury-induced inflammatory response have also been highly implicated in tumor progression when secreted by neutrophils in the TME (reviewed in [117]). Various soluble factors were reported to directly foster cancer cell growth and survival either via a direct activation of proliferation [118,119] or via counteracting senescence programs [120].

Neutrophils can contribute to the localized oxygen depletion when undergoing respiratory blast in response to acute intestinal inflammation and, in this case, the resulting microenvironmental hypoxia promoted inflammation resolution [32]. However, in other tissue contexts, a hypoxic environment was reported to increase degranulation and induce bronchial epithelial damage [121]. In the context of lung cancer, hypoxia-generating neutrophils promoted a pro-tumorigenic environment [122], but again, in a different tissue, hypoxia promoted neutrophils tumoricidal activity during uterine cancer initiation [123]. Therefore, the link between tissue hypoxia and neutrophil activity in both injury and cancer appears to be highly dependent on the tissue context, and likely also by the level of hypoxia itself.

Finally, an important function of neutrophils in inflammation resolution and repair is their ability to drive macrophage responses [10,124]. In cancer, both neutrophils and macrophages are an integral part of the tumor supportive TME and their presence can predict responses to immune checkpoint inhibitors [125], yet how these cells interact and influence each other in the TME require further investigations. Interestingly, the interaction between neutrophils and monocytes was reported to modulate the pro- vs anti- tumor activity in patient derived breast cancer xenografts [126], highlighting their potential to transition between states with opposing functions.

Overall, this distinct overlap between neutrophil functions in repair and cancer lends to the hypothesis that the pro-tumorigenic activity of neutrophils (or a subset of neutrophils) in the TME is guided by an intrinsic program of tissue repair, which becomes activated upon sensing a tissue perturbation when cancerous cells begin to outgrow. Importantly, this would suggest that a deeper understanding of the function of neutrophils in tissue healing may uncover new insights into their role in driving cancer progression and metastasis. Having a more holistic view of neutrophils in cancer inflammation, taking into account their response to other types of perturbations occurring in that tissue, could inform the development of approaches to target their pro-tumorigenic activities or even exploit their anti-tumor function.

## 5 What injury does to cancer

This parallelism between neutrophil activities in tissue repair and cancer promotion also raises the possibility that, in situations where a tissue injury occurs in the presence of a growing tumor, recruited neutrophils could inadvertently foster cancer progression. A direct causal link between tissue injury and cancer during tumor development remains relatively unchartered territory. However, in the context of the tumor disease when external insults are reported to foster cancer growth and colonization in secondary tissues, emerging experimental evidence suggests that neutrophil functions are key in bridging tissue injury and repair activity with tumor support.

Tobacco represents a chemical insult to the lung tissue and is a known cause of lung cancer. Tobacco smoke exposure following K-ras driven tumor initiation in mice had a tumor-promoting effect, via the induction of low-grade inflammation characterized by NF-κB-mediated production of IL-6 and TNF-α [127]. Importantly, myeloid-cell specific ablation of NF-κB signalling nearly completely abrogated the tobacco-smoke induced tumor promotion, reducing lung adenoma cell proliferation and tumor angiogenesis. Importantly, tobacco smoke was also reported to foster breast cancer metastatic growth in the lung [114]. Here neutrophils responded to LPS, a contaminant in tobacco smoke, by releasing NETs, which in turn induced ECM remodelling and the production of a pro-tumorigenic integrin activating laminin epitope [114].

In the context of the skin, UV-damaged epidermal keratinocytes were shown to trigger the recruitment and activation of neutrophils, dramatically influencing melanoma pathogenesis independent of tumor-initiation [128]. Neutrophils stimulated angiogenesis, which catalysed perivascular invasion and metastatic dissemination to the lungs. Thus, UV radiation not only induces tumor-initiating genomic alterations, but also promotes tumor progression and metastasis via neutrophil-dependent microenvironmental perturbations.

Cytotoxic chemotherapy and radiotherapy are important standard treatments against cancer with indisputable overall patient benefit. The phenomenon of collateral tissue damage they can cause represents a less-appreciated mechanism linking tissue injury and the pro-tumorigenic process, which may play a role in both therapy efficacy and resistance. This has been demonstrated in fibroblasts, where chemotherapy-induced DNA damage to the tumor stroma stimulates the production of soluble WNT16B and SPINKJ, which function as paracrine factors to promote tumor progression and attenuate chemotherapy-induced cytotoxicity [129,130]. Furthermore, healthy tissue exposure to ionizing radiation or systemic doxorubicin administration leads to elevated circulating levels of TGF-β1, which was directly linked to increased circulating tumor cells in mice bearing primary mammary tumors, and enhanced lung metastases [131]. Importantly, in the context of radiation injury, our laboratory recently demonstrated a direct link between injury-responding neutrophils and breast cancer metastasis to the lung [132]. Upon injury to healthy lung tissue, neutrophils locally acquire a pro-inflammatory phenotype with tissue perturbation functions that contribute to the overall tissue response. Particularly, radiation-primed neutrophils promoted the activation of regenerative Notch signalling in the lung epithelium, conditioning the tissue into a tumor-supportive microenvironment that ultimately increased metastatic growth. Importantly, the pro-metastatic effect of radiation-injury could be almost entirely abrogated by either neutrophil depletion, by inhibiting Notch signalling, or by the specific blocking of neutrophil degranulation, directly linking the role of neutrophils in the radiation-induced injury response to a tumor-supportive function.

## 6 Concluding remarks

Overall, at least some degree of neutrophil-mediated tissue damage is an inevitable and necessary by-product of tissue repair, ensuring a sufficient inflammatory response that ultimately restores tissue homeostasis. Impressively, organs may have evolved mechanisms to protect themselves, through inflammation resolution via macrophage efferocytosis, neutrophil disarming, and neutrophil temporal polarisation, allowing a dynamic switch in neutrophil phenotype from tissue-injuring to tissue-restoring. This neutrophil heterogeneity could perhaps explain the dichotomy of beneficial and detrimental roles of neutrophils in different injury contexts, analogous to the emerging reports of functionally distinct pro- and anti-tumoral neutrophil subsets in cancer. Whether the relative abundance of specialised neutrophil subsets can dramatically modulate the outcome of tissue healing remains a question of great interest in the field of regenerative medicine, as it could promote the development of therapies that drive neutrophil differentiation into pro-reparative phenotypes. In addition, the source of neutrophil heterogeneity in tissue healing remains unresolved, and it is not fully clarified whether functionally distinct neutrophils reflect their differential adaptation to local environments, or to systemic signals, or whether it reflects neutrophil intrinsic programs or, more likely a combination of all.

One of the aims of this review was to draw parallels between the activities of neutrophils in different tissue injury contexts, and their tumor-promoting roles in cancer. It is our view that an enhanced understanding of the precise cues that regulate the transition from physiological tissue repair to chronic tissue damage could provide new insights into the evolution of the tumor microenvironment during tumor outgrowth. Thus, understanding how neutrophils contribute to the switch between tissue injury and regeneration could uncover novel ways to restrain pro-tumoral inflammation and unleash anti-tumoral power.

## Data availability

This article did not involved any primary data.
